# Differences in gut microbiota composition between obese and lean children: a cross-sectional study

**DOI:** 10.1186/1757-4749-5-10

**Published:** 2013-04-30

**Authors:** Liene Bervoets, Kim Van Hoorenbeeck, Ineke Kortleven, Caroline Van Noten, Niel Hens, Carl Vael, Herman Goossens, Kristine N Desager, Vanessa Vankerckhoven

**Affiliations:** 1 Faculty of Pharmaceutical, Biomedical and Veterinary Sciences, University of Antwerp, Antwerp, Belgium; 2 Faculty of Medicine and Life Sciences, Hasselt University, Agoralaan 1-Building D, Diepenbeek, 3590, Belgium; 3 Laboratory of Experimental Medicine and Pediatrics, University of Antwerp, Antwerp, Belgium; 4 Department of Pediatrics, Antwerp University Hospital, Antwerp, Belgium; 5 Faculty of Medicine and Health Sciences, University of Antwerp, Antwerp, Belgium; 6 Center for Statistics, Hasselt University, Diepenbeek, Belgium; 7 Vaccine & Infectious Disease Institute, University of Antwerp, Antwerp, Belgium; 8 Department of Microbiology, Klina Hospital, Antwerp, Belgium

**Keywords:** Gut microbiota, 16S rDNA, MALDI-TOF MS, *Bacteroides fragilis* group, Obesity, Children

## Abstract

**Background:**

An altered gut microbiota composition has recently been linked to obesity. The principal aim of this study is to investigate and compare the gut microbiota composition in obese and lean children. Secondly, associations between analysed gut bacterial species, dietary compounds, energy intake and biochemical blood parameters are evaluated.

**Methods:**

In this prospective cross-sectional study, 26 overweight/obese (mean BMI: 28.7 ± 6.5) and 27 lean (mean BMI: 16.5 ± 2.1) children aged 6 to 16 were included. Faecal samples were collected and subjected to selective plating and quantitative real-time PCR (qPCR) in order to determine the concentrations of bacterial species belonging to the genera: *Bacteroides, Bifidobacterium*, *Clostridium*, *Staphylococcus* and *Lactobacillus*. Matrix-assisted laser desorption/ionization time-of-flight mass spectrometry (MALDI-TOF MS) was applied for an in-depth identification of species of *Bacteroides fragilis* group. Differences in the concentrations of gut bacterial species between obese and lean children were statistically analysed using Mann Whitney *U* test. Subsequently, random forest analysis and multiple linear regression analysis were performed in order to test associations between gut bacterial species, dietary compounds and blood parameters.

**Results:**

Obese children showed an elevated Firmicutes-to-Bacteroidetes ratio compared with lean children. Furthermore, low relative proportions of *B. vulgatus* and high concentrations of *Lactobacillus* spp. were observed in the obese microbiota. In all children, *Staphylococcus* spp. were positively associated with energy intake. Additionally, in obese children, *Lactobacillus* spp. were positively associated with plasma hs-CRP.

**Conclusions:**

Our findings corroborate a significant difference in the gut microbiota composition of important bacterial species between obese and lean children. In future, non-invasive manipulation of gut microbiota composition in early infancy could offer a new approach to manage childhood obesity and associated disorders.

## Background

Although there is evidence that the prevalence of childhood obesity is stabilising at different levels in different countries [1], the number of children and adolescents being overweight or obese is still dramatically high [2,3]. The major concern is that these children are at high risk of developing severe co-morbidities such as metabolic syndrome, non-alcohol fatty liver disease, type 2 diabetes mellitus and premature cardiovascular diseases [4,5]. Moreover, obese children are highly prone to become obese adults, especially when having a high body mass index (BMI) or an obese parent [6,7]. In order to combat childhood obesity and related complications, prevention is crucial. At the moment, the most important strategies to manage childhood obesity are therapeutic lifestyle changes, such as changing dietary habits and the physical activity level. However, these are often difficult to achieve. When lifestyle modifications continue to fail, pharmacological interventions and possibly bariatric surgery could be considered.

Nowadays, it is generally accepted that the development of obesity is caused by gene-environment interactions, generating a chronic positive energy balance [8]. However, physiological and environmental predispositions underlying obesity and associated metabolic disorders are still underexplored. Recent evidence suggests that our gut microbiota is involved in energy regulation as well as inflammation [9], and should therefore be considered as an environmental factor playing a role in the pathophysiology of obesity [10,11]. Although energy intake can affect the gut microbiota composition [12], it is still unclear whether the gut microbiota play a causal role in the development of obesity in humans.

So far, several studies in humans and mice have shown differences in gut microbiota composition between obese and lean subjects. These differences were mostly detected at the phylum level of mainly Firmicutes and Bacteroidetes [11-14]. Obesity in humans has already been associated with low intestinal concentrations of Bacteroidetes and high concentrations of Firmicutes, although this finding has been contradicted by other studies [15,16]. Only few studies have investigated the prevalence of faecal bacterial phyla in obese children and adolescents. One study demonstrated low concentrations of Bacteroidetes and high concentrations of Firmicutes in the distal gut of obese adolescents living in Spain [17]. Another study in Sweden, did not find significant differences in the concentrations of *Bacteroides fragilis* group, *Lactobacillus* spp. and *Bifidobacterium* spp. between preschool children with excessive body weight and normal-weight children [18]. By contrast, Vael et al. [19] demonstrated that a high intestinal concentration of *Bacteroides fragilis* group present in early infancy was associated with a higher risk of obesity later in life. In general, limited and contradictory findings with regard to the composition of the gut microbiota in obese children indicate that further in-depth analysis of the role of the intestinal microbiota in childhood obesity is warranted.

The principal aim of this study is to evaluate and compare the presence of certain gut bacterial species in faecal samples of obese and lean children and adolescents. Quantitative culturing was used to identify and determine the concentrations of the following bacterial genera: *Bacteroides fragilis* group, *Bifidobacterium*, *Clostridium*, *Staphylococcus* and *Lactobacillus*. In addition to quantitative culturing, matrix-assisted laser desorption/ionization mass spectrometry (MALDI-TOF MS) was used for in-depth analysis of species belonging to the *Bacteroides fragilis* group. Quantitative real-time polymerase chain reaction (qPCR) was applied to quantify *Bacteroides-Prevotella-Porphyromonas* spp.*, Bifidobacterium* spp.*, Clostridium coccoides-Eubacterium rectale* group*, Clostridium leptum* group*, Staphylococcus* spp. and *Lactobacillus* spp. The Firmicutes-to-Bacteroidetes ratio was calculated based on the qPCR results. Finally, analysed gut bacterial species were associated with dietary compounds and energy intake, which were assessed by dietary records. Moreover, concentrations of biochemical blood parameters were measured in overweight and obese subjects.

## Results

### Subject characteristics

Characteristics of the study population are shown in Table 1. In total, 9 overweight, 7 obese, 10 morbidly obese (O/O) children and 21 normal-weight, 5 thinness grade I and 1 thinness grade III (C) children were included (see Methods section for details on BMI classification). Age, gender, height and dietary intake were not significantly different between the two study groups.

**Table 1:** General characteristics of the studied population

	**O/O (n = 26)**	**C (n = 27)**	**p value**
**Anthropometric data**			
Gender (F/M), n	12/14	11/16	0.691
Age, y	11.64 ± 2.43	10.70 ± 3.12	0.229
Height, cm	153.6 ± 15.7	144.7 ± 18.6	0.063
Weight, kg	69.8 ± 25.1	35.7 ± 12.6	< 0.0001*
BMI, kg/m^2^	28.73 ± 6.53	16.48 ± 2.10	< 0.0001*
BMI SDS	2.69 ± 0.80	−0.43 ± 0.96	< 0.0001*
**Dietary data**			
Number, n	22	25	
Energy, kcal/d	2 231.95 ± 437.69	2 082.32 ± 446.79	0.254
Carbohydrates, en%	45.68 ± 9.55	48.81 ± 6.28	0.199
Fat, en%	40.08 ± 9.73	38.59 ± 6.01	0.537
Protein, en%	14.43 ± 3.94	14.48 ± 6.54	0.976
Dietary fibre, g/d	19.26 ± 8.33	15.64 ± 7.33	0.120

O/O: obese group; C: control group; BMI SDS: body mass index standard deviation score. *Significant differences with p value < 0.05.

### Quantification of bacterial genera and species of *Bacteroides fragilis* group

**Figure 1:**
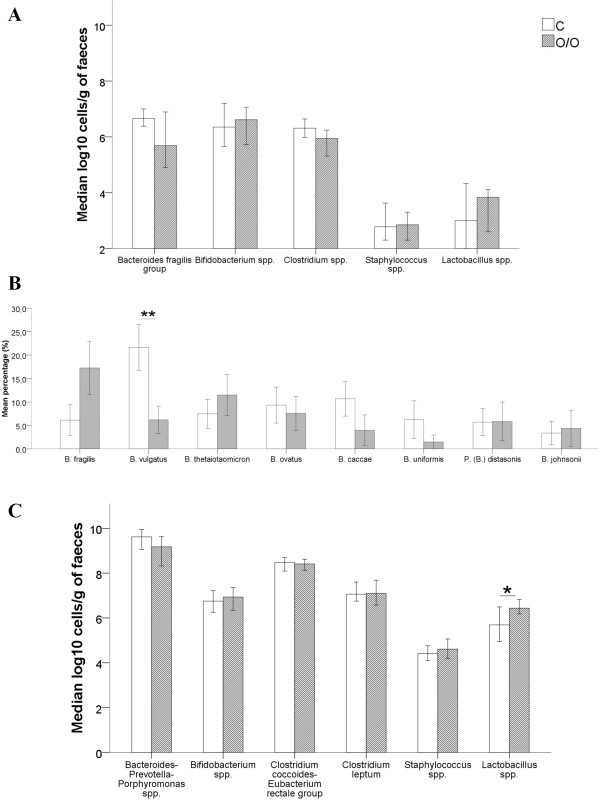
**Differences in bacterial genera between O/O and C group. A**: Differences in bacterial genera between O/O and C detected by quantitative plating. **B**: Differences in relative proportions of *Bacteroides fragilis* group species between O/O and C detected by MALDI-TOF MS. **C**: Differences in bacterial genera between O/O and C detected by qPCR. Data of quantitative plating and qPCR are expressed as mean log_10_ cells/g of faeces. Data of MALDI-TOF MS are reported in percentages (%). O/O: obese group; C: control group. Error bars 95% CI. **p = 0.004. *p = 0.04.

Differences in the concentrations of bacterial genera between O/O and C children are presented in Figure 1A, B and C. Figure 1A illustrates differences between gut microbiota in O/O and C children detected by quantitative plating. *Bacteroides fragilis* group and *Clostridium* spp. were borderline, but non-significantly different between O/O and C children (5.69 ± 2.14 vs. 6.66 ± 0.84 and 5.94 ± 1.10 vs. 6.31 ± 0.80, respectively; p = 0.050 and p = 0.074). In-depth analysis of species belonging to the *Bacteroides fragilis* group using MALDI-TOF MS revealed dominating relative proportions of *B. fragilis* (17.3% vs. 6.1%, p = 0.136) and *B. thetaiotaomicron* (11.5% vs. 7.5%, p = 0.930) in faecal samples of O/O children compared with C children (Figure 1B). By contrast, in C children, relative proportions of *B. caccae* (10.7% vs. 4.0%, p = 0.051), *B. ovatus* (9.3% vs. 7.6%, p = 0.585), *B. uniformis* (6.3% vs. 1.5%, p = 0.177) and *B. vulgatus* (21.7% vs. 6.2%, p = 0.004) prevailed. Note that only *B. vulgatus* proportions were significantly different between the O/O and C children. Figure 1C shows differences found between gut bacterial species in O/O and C children detected by qPCR. In contrast to the quantitative plating results, faecal concentrations of *Lactobacillus* spp. were found to be significantly higher in O/O compared with C children and adolescents (6.44 ± 1.20 vs. 5.69 ± 1.80, p = 0.035) using qPCR.

### The Firmicutes-to-Bacteroidetes ratio

**Figure 2:**
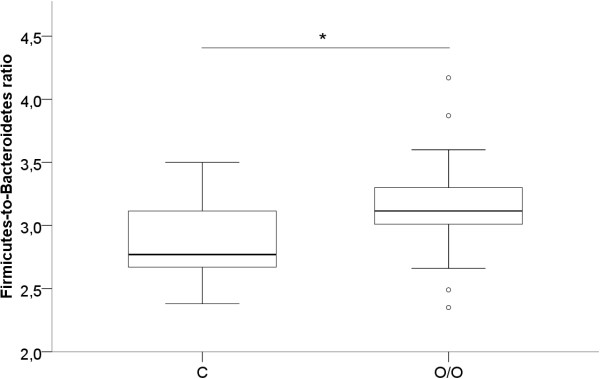
**Firmicutes-to-Bacteroidetes ratio of O/O versus C children.** O/O: obese group; C: control group. *p = 0.007.

In Figure 2, a boxplot of the Firmicutes-to-Bacteroidetes ratio of O/O and C children is presented. The ratio resulted in favour of the Firmicutes in O/O children and adolescents (p = 0.007).

### Dietary assessment

The most important associations between analysed gut bacterial species, dietary compounds and energy intake in a subsample of 22 O/O and 25 C children are presented in Table 2. Children and adolescents with a high daily energy intake showed high faecal concentrations of *Staphylococcus* spp., analysed by means of quantitative plating (p = 0.028).

**Table 2:** Most important associations between gut microbiota and dietary compounds represented by regression coefficient β (p value)

	**Dietary compounds**				
	**Carbohydrates (en%)**	**Fat (en%)**	**Protein (en%)**	**Fibre (g/d)**	**Energy intake (kcal/d)**
**Quantitative plating**					
*Bacteroides fragilis* group	0.127 (0.110)	0.129 (0.129)	0.001 (0.987)		−0.000 (0.620)
*Bifidobacterium* spp.	−0.025 (0.410)				0.000 (0.822)
*Clostridium* spp.					0.001 (0.156)
*Staphylococcus* spp.					0.001 (0.028)*
*Lactobacillus* spp.	0.069 (0.360)	0.104 (0.180)			
**qPCR**					
*Bacteroides-Prevotella-Porphyromonas* spp.	0.048 (0.319)	0.069 (0.220)			−0.000 (0.830)
*Bifidobacterium* spp.	0.017 (0.439)				0.000 (0.285)
*Clostridium coccoides-Eubacterium rectale* group					0.000 (0.269)
*Clostridium leptum* group					0.000 (0.240)
*Staphylococcus* spp.	−0.093 (0.203)	−0.084 (0.285)	−0.083 (0.075)		0.000 (0.691)
*Lactobacillus* spp.	0.052 (0.503)	0.069 (0.401)	−0.000 (0.993)	0.010 (0.745)	0.000 (0.415)

Regression coefficient with 95% CI. *Significant association with p value < 0.05.

### Biochemical markers

Important biochemical markers were measured in fasting venous blood samples of 19 obese children (see Material and Method section for more details on blood sampling procedure). The following mean values were obtained: fasting plasma glucose: 82.44 ± 5.34 mg/dl; fasting plasma insulin: 21.58 ± 17.31 μU/ml; total cholesterol (TC): 176.26 ± 42.14 mg/dl; high-density lipoprotein (HDL) cholesterol: 49.61 ± 10.05 mg/dl; triglycerides (TG): 112.63 ± 81.92 mg/dl; leukocytes: 7.86 ± 2.42%; high-sensitive C-reactive protein (hs-CRP): 0.46 ± 0.36 mg/dl; alanin aminotransferase (ALT): 38.12 ± 19.82 U/l; aspartate aminotransferase (AST): 33.94 ± 12.79 U/l. The most important associations between major gut microbiota species and the concentration of the biochemical markers are presented in Table 3. Intestinal concentrations of *Lactobacillus* spp., which were analysed by quantitative plating, showed a positive association with plasma hs-CRP levels (p = 0.007).

**Table 3:** Most important associations between biochemical parameters, gut microbiota and BMI SDS represented by regression coefficient β (p value)

	**Biochemical markers**								
	**Leukocytes**	**Glucose**	**Insulin**	**HDL-C**	**TG**	**TC**	**ALT**	**AST**	**hs-CRP**
	**(%)**	**(mg/dl)**	**(μU/ml)**	**(mg/dl)**	**(mg/dl)**	**(mg/dl)**	**(U/l)**	**(U/l)**	**(mg/l)**
**Quantitative plating**									
*Bacteroides fragilis* group		−1.469		−1.677		−6.926			
		(0.237)		(0.099)		(0.497)			
*Bifidobacterium* spp.		0.830				5.673	−1.619	−0.124	
		(0.631)				(0.719)	(0.753)	(0.969)	
*Clostridium* spp.	−0.276	0.071	−3.725		2.322	22.353		−3.598	
	(0.562)	(0.967)	(0.343)		(0.932)	(0.181)		(0.245)	
*Staphylococcus* spp.				1.718	−30.933	−32.137			
				(0.414)	(0.381)	(0.122)			
*Lactobacillus* spp.			2.040						0.171
			(0.497)						(0.007)*
**qPCR**									
*Bacteroides-Prevotella-Porphyromonas* spp.				−0.668					
				(0.771)					
*Bifidobacterium* spp.									
*Clostridium coccoides-Eubacterium rectale* group	0.860	0.825		1.259		33.654	−3.686	−6.465	0.285
	(0.107)	(0.810)		(0.688)		(0.234)	(0.518)	(0.189)	(0.292)
*Clostridium leptum* group									
*Staphylococcus* spp.					11.856				
					(0.481)				
*Lactobacillus* spp.		−1.307	1.342		17.604	−5.764		6.274	−0.004
		(0.518)	(0.703)		(0.468)	(0.717)		(0.139)	(0.979)
**BMI SDS**	0.726	2.449	1.008	2.588	75.925	29.824	19.310	10.655	0.181
	(0.469)	(0.392)	(0.900)	(0.406)	(0.086)	(0.207)	(0.072)	(0.117)	(0.390)

Regression coefficient with 95% CI. *Significant association with p value < 0.05.

## Discussion

At birth, the gut microbiota of an infant is sterile but rapidly assembles over days or months [20]. Mode of delivery (natural delivery versus caesarean section) and feeding method (breast feeding versus bottle feeding) have an early impact on the development of a child’s gut microbiome [21]. At the age of four, the gut microbiota is fully mature [22]. Eventually, each person develops a unique gut microbiota which is stable over time in healthy adults [23].

In this cross-sectional study, the obese gut microbiota composition was compared with that of a lean one. We focused on two major phyla Bacteroidetes and Firmicutes, next to the *Bacteroides fragilis* group, *Bifidobacterium, Clostridium*, *Staphylococcus* and *Lactobacillus*. Different bacterial groups were selected according to the frequency to which they have been described in relevant literature [17-19,24] and the ease of detection by the techniques used. On the one hand, quantitative plating was used as the ‘gold standard’ technique to isolate and characterise the selected bacterial groups. However, only 10 to 50% of all bacteria associated with the human body can be cultivated successfully [23,25]. Subsequently, high-throughput culture-independent techniques, which use DNA sequences encoding the 16S ribosomal RNA subunit, were applied in order to assign an organism to a phylogenetic classification more accurately [25].

To our knowledge, our study was the first to perform an in-depth analysis of species belonging to the *Bacteroides fragilis* group by means of MALDI-TOF MS. Overall, our results reveal a high Firmicutes-to-Bacteroidetes ratio in faeces of obese children including alterations at species level.

Selective media have been used successfully to identify and enumerate *Bacteroides fragilis* group from human faeces [26]. For the first time, a further in-depth analysis of species of the *Bacteroides fragilis* group revealed reduced relative proportions of *B. vulgatus* in obese children and adolescents. One study reported decreased relative proportions of *B. vulgatus* in the faeces of type 2 diabetic subjects using species specific PCR-denaturing gradient gel electrophoresis (DGGE) [27]. *B. vulgatus* was found to constitute a part of the core gut microbiota in healthy humans and is generally considered to be a beneficial gut commensal [28]. These findings point towards a possible role for *B. vulgatus* in the pathophysiology of Western diseases, such as obesity and diabetes.

Moreover, the qPCR method that was used in this study to detect and quantify Bacteroidetes (*Bacteroides-Prevotella-Porphyromonas* spp.)*,* Firmicutes (*Clostridium coccoides-Eubacterium rectale* group*, Clostridium leptum* group*, Staphylococcus* spp. and *Lactobacillus* spp.), and *Bifidobacterium* spp. in human faeces has already been thoroughly evaluated and validated [29-31]. In agreement with the findings of previous studies [32,33], we describe higher concentrations of *Lactobacillus* spp. in the obese gut microbiota. However, the use of quantitative plating did not permit the detection of a significantly higher concentration of *Lactobacillus* spp. in faeces of obese children, which we did see using qPCR. A possible explanation is that *L. gasseri* and *L. acidophilus* could not be identified in culture due to the presence of vancomycin in the LAMVAB medium [34]. Nevertheless, both quantitative culturing and qPCR resulted in a similar proportion of *Lactobacillus* spp. in the obese gut microbiota. A study conducted by Million et al. [32], demonstrated that *Lactobacillus reuteri* was associated with obesity in adults. By contrast, Santacruz et al. [33] showed that BMI SDS reduction in obese adolescents led to a concomitant increase in the concentrations of *Lactobacillus* spp. These findings thus suggest a possible role of *Lactobacillus* at species level in body weight and obesity. Additionally, we showed that the concentration of *Lactobacillus* spp. is positively correlated to plasma hs-CRP levels in obese children and adolescents. An increased prevalence of positive Firmicutes to higher levels of plasma hs-CRP was also seen in a study conducted in 51 obese and 28 normal-weight children and adults [35]. These results seems therefore to suggest a possible role for *Lactobacillus* spp. in “low-grade” inflammation, a major pathophysiological process of obesity.

Interestingly, we detected an elevated Firmicutes-to-Bacteroidetes ratio in the gut microbiota of obese children and adolescents. Previous investigators also showed significant associations between this ratio and obesity in mice and humans [11-14]. The results of our study are similar to a study in Spanish children, demonstrating increased concentrations of Firmicutes and decreased concentrations of Bacteroidetes in the obese gut [17]. Contrary to these findings, other studies described no or even opposite differences in the Firmicutes-to-Bacteroidetes ratio between obese and lean subjects [15,16]. Possibly, these variations in study outcome are related to the fact that different methodologies were applied in these studies.

To further elucidate the complex role of gut microbiota in host physiology, a more thorough examination of the influence of diet on gut microbiota is recommended. In order to do so, we analysed the relationship between the presence of certain gut bacterial species with dietary compounds and energy intake. Here, we demonstrate that, independent of the BMI status, children and adolescents with a high energy intake (expressed in kcal/d) possess high faecal concentrations of *Staphylococcus* spp. analysed by quantitative culture. Note that the regression coefficient β of energy intake is low in all cases. This is due to the fact that values of energy intake are expressed in kcal/d. Given the range of energy intake (1635.53 to 2669.64 kcal), results in effect on mean concentration of *Staphylococcus* spp. of 1.27 to 2.08 are obtained. These results are not negligible and a real significant association has been detected. However, caution must be taken when translating these findings into a biological meaningful interpretation. Hence, more detailed research on this topic is necessary. Nevertheless, the importance of *Staphylococcus* spp. in childhood obesity has already been demonstrated by Kalliomaki et al. [24] who showed that a greater faecal concentration of *Staphylococcus* spp. during infancy predicted the development of overweight during childhood. A possible role of *Staphylococcus* spp. in energy harvesting during childhood is thus suggested.

One major limitation of the current study is the small sample size and therefore these results should be interpreted with caution. In addition, pregnancy related factors, social status, and the period of being obese prior to inclusion were not taken into account.

Further longitudinal research on the cause-effect relationship between gut microbiota and obesity is highly justified, since different bacterial species could play a significant role in the human energy harvest and weight regulation. Moreover, consideration of lifestyle factors in gut microbiota studies is highly recommended, since changes in dietary pattern and physical activity could influence gut microbiota composition and the development of obesity. Finally, we suggest to focus future research not only on the elucidation of gut microbiota composition in obese subjects, but also on the study of gut metabolites, i.e. “metabolomics”. This suggestion for future research aims at expanding our knowledge on the complex interplay between gut microbiota, energy homeostasis and obesity.

In the future, modification of the gut microbiota composition by the administration of pro-, pre- or synbiotics in early childhood could offer an opportunity to prevent and/or treat obesity [36]. However, additional research is required.

## Conclusions

In this study, important compositional differences in the faecal gut microbiota of obese and lean children were revealed. This was generally reflected by an elevated Firmicutes-to-Bacteroidetes ratio in the obese study population. At the species level, low proportions of *B. vulgatus* and high concentrations of *Lactobacillus* spp. were found in faeces of obese children and adolescents. Furthermore, the presence of *Lactobacillus* spp. in the obese gut microbiota was positively associated with plasma hs-CRP levels. We also found a positive association between energy intake and the presence of *Staphylococcus* spp. in faeces of children, independent of their BMI status. The aforementioned bacterial genera and species may thus be more efficient at extracting energy from a given diet in obese children and adolescents compared with gut microbiota of lean children. Hence, *B. fragilis* group, *Lactobacillus* spp. and *Staphylococcus* spp. play an important role in the pathophysiology of childhood obesity. We hypothesize that an aberrant gut microbiota composition in combination with influences of lifestyle factors might contribute to the development of childhood obesity. To further confirm this hypothesis, additional research is required in a longitudinal setting with recruitment of a larger sample population. Finally, prevention and treatment strategies based on modification of the gut microbiota in obese children might conceivably contribute to limit the development of future obesity.

## Methods

### Subject characteristics

Overweight, obese and morbidly obese (O/O: obese group) children were recruited from the paediatric obesity clinic at the Antwerp University Hospital. Normal-weight and lean children (C: control group) were recruited among the offspring of personnel working at the University of Antwerp. Skilled personnel recorded weight (scale, SECA 701 scale, Hamburg, Germany) and height (stadiometer, SECA 225, Hamburg, Germany) measurements, to the nearest 0.1 kg for weight and 1 mm for height and the BMI (in kg/m^2^). All subjects were classified based on the international BMI cut-off values of the Extended International Obesity Task Force (IOTF) for children aged 2 to 18 [37]. These values are based on age and gender-specific centile curves passing through BMI 35 (morbid obesity), 30 (obesity), 25 (overweight) and 18.5, 17 and 16 (thinness grades I, II and III) at age 18. BMI standard deviation score (SDS) was assessed using an electronic calculator (Auxology 1.1, Pfizer, New York, USA) based on the local reference standards, i.e. Flemish growth charts [38]. Exclusion criteria included the use of corticosteroids or antibiotics in the month prior to the study as well as during the study and significant co-morbidities such as an acute infection, prematurity or chronic diseases. The study was conducted in accordance with the ethical rules of the Helsinki Declaration. Informed consent was obtained from all children and their parents or legal guardian. The study protocol was approved by the local medical Ethics Committee of the Antwerp University Hospital (document 7/41/226).

### Gut microbiota analyses

#### Faecal sample collection

A fresh faecal sample was self-collected and stored immediately at −20°C. The collection took place in the same week as the dietary record. Subsequently, the faecal samples were transported to the laboratory and stored at −80°C until further analysis.

#### Quantitative plating

Approximately 0.5 g of wet faeces was diluted in 9 volumes of phosphate buffer saline (PBS) and homogenized using a stomacher (Minimix®, Interscience, Arpents, France). Serial dilutions were plated using the Eddy Jet® apparatus (Led Techno, Heusden-Zolder, Belgium) onto the selective growth media. More specifically, *Staphylococcus* spp. were cultivated on mannitol salt agar (MSA) (Becton-Dickinson, Erembodegem, Belgium); only yellow and white colonies were considered [39]. *Bacteroides fragilis* group was determined on *Bacteroides* Bile Esculin agar (BBE) (Becton-Dickinson, Erembodegem, Belgium); only black pigmented colonies were considered [40]. *Clostridium* spp. counts were obtained after pre-treatment of the faecal sample in 70% ethanol for 30 minutes and subsequent culture on Columbia blood agar (CBA) [41]. *Lactobacillus* spp. were cultured on *Lactobacillus* anaerobic de Man, Rogosa and Sharpe with vancomycin and bromocresolgreen (LAMVAB) medium; only green and white colonies were considered [34]. *Bifidobacterium* spp. were determined on modified trypticase-phytone-yeast agar (MTPY); only colonies smaller than 0.7 mm were considered [42]. To confirm the presence of *Bifidobacterium* spp. in these colonies, a gram stain was conducted. The numbers of colonies positive for gram stain were expressed relative to the total number of colonies examined.

#### MALDI-TOF MS analysis

Of each sample, ten presumed colonies of *Bacteroides fragilis* group were subcultured on Wilkins-Chalgren medium for 24h and spotted onto a target plate. Matrix-assisted laser desorption/ionization mass spectrometry (MALDI-TOF MS) analysis was accomplished according to the manufacturer’s instructions (Microflex™ LT Bruker Daltonik GmbH, Bremen, Germany). Recorded mass spectra generated with the MALDI Biotyper 2.0 software package were compared with each entry of the MALDI Biotyper database and relationships were identified. According to the criteria by Nagy et al. [26], only identifications with a log (score) between 1.9 and 3.0 were considered to be significant and allowed for identification of the bacterial populations at the species level.

#### Quantitative real-time PCR

##### DNA extraction

An aliquot (200 μl) of faecal suspension was suspended in 300 μl Tris-SDS solvent, 300 μg glass beads (diameter, 0.1 mm) and 500 μl TE-saturated phenol. Further extraction of total bacterial DNA was performed as described by Matsuki et al. [30]. The mixture was vortexed vigorously with FastPrep® FP120A (BIO 101® Systems, Thermo Scientific) for 30 s at a power level of 5.0. After centrifugation for 5 min at 13 000 rpm, 400 μl of the supernatant was collected and 400 μl phenol/chloroform/isoamylalcohol (25:24:1) was added. Subsequently, 250 μl of the supernatant was subjected to isopropanol.

##### qPCR

qPCR assay was performed as previously described by Rinttilä et al. [31]. All PCR experiments were carried out in triplicate with a reaction volume of 25 μl. The reaction mixture contained 7.5 μl sterile Milli-Q; 12.5 μl iQ SYBR Green Supermix and 1.25 μl of 10 μM of each primer (forward and reverse). Subsequently, 2.5 μl of faecal DNA was added to the reaction mixture. For each bacterial strain, positive controls were added to each reaction and the total bacterial copy number per organism was determined with 16S rRNA gene targeted primers [29-31]. The bacterial primer sequences are reported in Table 4.

**Table 4:** 16S rRNA gene-targeted group specific primers per bacterial group/species used in this study

**Target**	**Primer sequence (5’-3’)**	**Reference strains**	**Annealing temp (°C)**	**Reference**
**Bacteroidetes**				
*Bacteroides-Prevotella-Porphyromonas* spp.	GGTGTCGGCTTAAGTGCCAT	*Bacteroides fragilis* DSM 2151/LMG 10263	68	[31]
	CGGA(C/T)GTAAGGGCCGTGC			
**Firmicutes**				
*Staphylococcus* spp.	GCGATTGATGGTGATACG	*Staphylococcus aureus* ATCC 29213	55	[29]
	AGCCAAGCCTTFACGAACTAAAGC			
*Clostridium coccoides-Eubacterium* rectale group (clostridial cluster XIV)	CGGTACCTGACTAAGAAGC	*Ruminococcus productus* DSM 2950/YIT 6141	55	[31]
	AGTTT(C/T)ATTCTTGCGAACG			
*C. leptum* group (clostridial cluster IV)	GCACAAGCAGTGGAGT	*Faecalibacterium prausnitzii* YIT 6174	50	[30]
	TTCCTCCGTTTTGTCAA			
*Lactobacillus* spp.	AGCAGTAGGGAATCTTCCA	*Lactobacillus acidophilus* LMG 9433	58	[31]
	CACCGCTACACATGGAG			
**Actinobacteria**				
*Bifidobacterium* spp.	TCGCGTC(C/T)GGTGTGAAAG	*Bifidobacterium longum* DSM 20219/YIT 4021	58	[31]
	CCACATCCAGC(A/G)TCCAC			

##### The Firmicutes-to-Bacteroidetes ratio

An estimation of the total amount of Firmicutes was obtained by adding bacterial values (in log_10_ cells/g faeces) from *Clostridium coccoides-Eubacterium* rectale group (clostridial cluster XIV).

*Clostridium leptum* group (clostridial cluster IV), *Lactobacillus* spp. and *Staphylococcus* spp. For the total amount of Bacteroidetes, the *Bacteroides-Prevotella-Porphyromonas* spp. were taken into consideration. The ratio was calculated according to Mariat et al. [43].

### Dietary assessment

A total of 22 (84.6%) O/O and 25 (92.6%) C children fully completed a five-day dietary record (three weekdays and two weekend days). The average daily intake of energy and nutrients was calculated with the use of Becel Institute Nutritional Software program. The dietary variables examined in this study were carbohydrates (energy %), fat (energy %), protein (energy %), fibres (g/day), and total energy intake (kcal/day).

### Biochemical analyses

Blood sampling was initially conducted to detect associated metabolic and inflammatory complications in the O/O group. Since only 19 out of 26 fasting venous blood samples of obese children were available, results of only these samples were considered. Glucose, total cholesterol (TC), high-density lipoprotein cholesterol (HDL-C), triglycerides (TG), alanine aminotransferase (ALT), aspartate aminotransferase (AST) and high-sensitive C-reactive protein (hs-CRP) were measured on Dimension Vista 1500 System (Siemens Healthcare Diagnostics Inc., Neward, Delaware, USA). Insulin levels were measured using chemoluminescence (Roche Diagnostics, Rotkreuz, Switzerland). White blood cell count was performed using flow cytometry (Advia 2120, Siemens Healthcare Diagnostics Inc., Neward, Delaware, USA).

### Statistical analysis

Descriptive and comparative analyses were performed in IBM SPSS version 20.0 (SPSS Inc., Chicago, IL, USA). The distribution of the residuals was tested for normality using the Kolmogorov-Smirnov test with Lilliefors correction. Independent samples *t* test was used in case of no significant deviation from normality. Otherwise the Mann Whitney *U* test was used. Chi square (*χ*^2^) test of association was used to compare characteristics between the O/O and C study groups. Data were presented as mean with standard deviation (mean ± SD) unless indicated otherwise. Since bacterial counts followed a right-skewed distribution, data were log_10_-transformed. Bacterial data were expressed as median log_10_ cells/g of faeces with interquartile ranges (IQR). Regression analyses were implemented in R 2.13.1. Explanatory variables were selected on the basis of a random forest analysis, i.e. a nonparametric technique that facilitates the selection of the important variables in a regression setting [44]. Given the random forest variable importance plot, the most important predictors above a visually selected cut-off value were chosen. Subsequently, multiple linear regression was applied to quantify the associations between gut microbiota, diet, biochemical parameters and BMI SDS controlled for age and gender. Wilcoxon-signed rank test was used to evaluate the difference of accuracy between quantitative culturing and qPCR. Statistical significance was assessed at the 5% level.

## Abbreviations

ALT: Alanin aminotransferase; AST: Aspartate aminotransferase; BBE: *Bacteroides* Bile Esculin; BMI SDS: Body Mass Index Standard Deviation Score; C: Control group; CBA: Columbia Blood Agar; HDL: High-density lipoprotein; hs-CRP: High sensitive C-reactive protein; IOTF: International Obesity Task Force; IQR: Inter Quartile Ranges; LAMVAB: *Lactobacillus* Anaerobic de Man Rogosa and Sharpe with Vancomycin And Bromocresolgreen; MALDI-TOF MS: Matrix-Assisted Laser-Desorption/Ionization Time-Of-Flight Mass Spectrometry; MSA: Mannitol Salt Agar; MTPY: Modified Trypticase-Phytone-Yeast Agar; O/O: Obese group; PBS: Phosphate Buffer Saline; TC: Total cholesterol; TG: Triglycerides; qPCR: Quantitative real-time polymerase chain reaction

## Competing interests

All authors declare that there are no competing financial interests in relation to the work described.

## Authors’ contributions

KD, CV, HG and VVK conceived and designed the study. LB, KVH, IK and CVN collected the data. LB carried out experiments. LB, IK, CVN and NH statistically analysed the data. LB, KVH, NH and VVK interpreted the data. LB, IK and CVN did literature research. LB generated figures and tables. LB wrote the manuscript with help of KVH and VVK. KVH, NH, KD, CV, HG and VVK revised the paper. LB had full access to all of the data in the study and takes responsibility for the integrity of the data and accuracy of the data analysis. All authors had final approval of the submitted and published versions.

## References

[B1] Olds T Maher C Zumin S Peneau S Lioret S Castetbon K Evidence that the prevalence of childhood overweight is plateauing: data from nine countries Int J Pediatr Obes 2011 6 342 360 10.3109/17477166.2011.605895 21838570

[B2] Gupta N Goel K Shah P Misra A Childhood obesity in developing countries: epidemiology, determinants, and prevention Endocr Rev 2012 33 48 70 10.1210/er.2010-0028 22240243

[B3] Lakshman R Elks CE Ong KK Childhood obesity Circulation 2012 126 1770 1779 10.1161/CIRCULATIONAHA.111.047738 23027812 PMC3785130

[B4] Weiss R Metabolic syndrome in childhood - causes and effects Endocr Dev 2010 19 62 72 20551669 10.1159/000316898

[B5] Burgert TS Taksali SE Dziura J Goodman TR Yeckel CW Papademetris X Alanine aminotransferase levels and fatty liver in childhood obesity: associations with insulin resistance, adiponectin, and visceral fat J Clin Endocrinol Metab 2006 91 4287 4294 10.1210/jc.2006-1010 16912127

[B6] Whitaker RC Wright JA Pepe MS Seidel KD Dietz WH Predicting obesity in young adulthood from childhood and parental obesity N Engl J Med 1997 337 869 873 10.1056/NEJM199709253371301 9302300

[B7] Freedman DS Khan LK Serdula MK Dietz WH Srinivasan SR Berenson GS The relation of childhood BMI to adult adiposity: the Bogalusa Heart Study Pediatrics 2005 115 22 27 15629977 10.1542/peds.2004-0220

[B8] Maffeis C Aetiology of overweight and obesity in children and adolescents Eur J Pediatr 2000 159 Suppl 1 S35 S44 11011954 10.1007/pl00014361

[B9] Cani PD Bibiloni R Knauf C Waget A Neyrinck AM Delzenne NM Changes in gut microbiota control metabolic endotoxemia-induced inflammation in high-fat diet-induced obesity and diabetes in mice Diabetes 2008 57 1470 1481 10.2337/db07-1403 18305141

[B10] Backhed F Ding H Wang T Hooper LV Koh GY Nagy A The gut microbiota as an environmental factor that regulates fat storage Proc Natl Acad Sci U S A 2004 101 15718 15723 10.1073/pnas.0407076101 15505215 PMC524219

[B11] Turnbaugh PJ Ley RE Mahowald MA Magrini V Mardis ER Gordon JI An obesity-associated gut microbiome with increased capacity for energy harvest Nature 2006 444 1027 1031 10.1038/nature05414 17183312

[B12] Murphy EF Cotter PD Healy S Marques TM O'Sullivan O Fouhy F Composition and energy harvesting capacity of the gut microbiota: relationship to diet, obesity and time in mouse models Gut 2010 59 1635 1642 10.1136/gut.2010.215665 20926643

[B13] Ley RE Backhed F Turnbaugh P Lozupone CA Knight RD Gordon JI Obesity alters gut microbial ecology Proc Natl Acad Sci U S A 2005 102 11070 11075 10.1073/pnas.0504978102 16033867 PMC1176910

[B14] Ley RE Turnbaugh PJ Klein S Gordon JI Microbial ecology: human gut microbes associated with obesity Nature 2006 444 1022 1023 10.1038/4441022a 17183309

[B15] Duncan SH Lobley GE Holtrop G Ince J Johnstone AM Louis P Human colonic microbiota associated with diet, obesity and weight loss Int J Obes (Lond) 2008 32 1720 1724 10.1038/ijo.2008.155 18779823

[B16] Schwiertz A Taras D Schafer K Beijer S Bos NA Donus C Microbiota and SCFA in lean and overweight healthy subjects Obesity (Silver Spring) 2010 18 190 195 10.1038/oby.2009.167 19498350

[B17] Ferrer M Ruiz A Lanza F Haange SB Oberbach A Till H Microbiota from the distal guts of lean and obese adolescents exhibit partial functional redundancy besides clear differences in community structure Environ Microbiol 2013 15 211 226 10.1111/j.1462-2920.2012.02845.x 22891823

[B18] Karlsson CL Onnerfalt J Xu J Molin G Ahrne S Thorngren-Jerneck K The microbiota of the gut in preschool children with normal and excessive body weight Obesity (Silver Spring) 2012 20 2257 2261 10.1038/oby.2012.110 22546742

[B19] Vael C Verhulst SL Nelen V Goossens H Desager KN Intestinal microflora and body mass index during the first three years of life: an observational study Gut Pathog 2011 3 8 10.1186/1757-4749-3-8 21605455 PMC3118227

[B20] Mackie RI Sghir A Gaskins HR Developmental microbial ecology of the neonatal gastrointestinal tract Am J Clin Nutr 1999 69 1035S 1045S 10232646 10.1093/ajcn/69.5.1035s

[B21] Penders J Thijs C Vink C Stelma FF Snijders B Kummeling I Factors influencing the composition of the intestinal microbiota in early infancy Pediatrics 2006 118 511 521 10.1542/peds.2005-2824 16882802

[B22] Vrieze A Holleman F Zoetendal EG de Vos WM Hoekstra JB Nieuwdorp M The environment within: how gut microbiota may influence metabolism and body composition Diabetologia 2010 53 606 613 10.1007/s00125-010-1662-7 20101384 PMC2830587

[B23] Vanhoutte T Huys G Brandt E Swings J Temporal stability analysis of the microbiota in human feces by denaturing gradient gel electrophoresis using universal and group-specific 16S rRNA gene primers FEMS Microbiol Ecol 2004 48 437 446 10.1016/j.femsec.2004.03.001 19712312

[B24] Kalliomaki M Collado MC Salminen S Isolauri E Early differences in fecal microbiota composition in children may predict overweight Am J Clin Nutr 2008 87 534 538 18326589 10.1093/ajcn/87.3.534

[B25] Zoetendal EG Rajilic-Stojanovic M de Vos WM High-throughput diversity and functionality analysis of the gastrointestinal tract microbiota Gut 2008 57 1605 1615 10.1136/gut.2007.133603 18941009

[B26] Nagy E Maier T Urban E Terhes G Kostrzewa M Species identification of clinical isolates of Bacteroides by matrix-assisted laser-desorption/ionization time-of-flight mass spectrometry Clin Microbiol Infect 2009 15 796 802 10.1111/j.1469-0691.2009.02788.x 19438622

[B27] Wu X Ma C Han L Nawaz M Gao F Zhang X Molecular characterisation of the faecal microbiota in patients with type II diabetes Curr Microbiol 2010 61 69 78 10.1007/s00284-010-9582-9 20087741

[B28] Qin J Li R Raes J Arumugam M Burgdorf KS Manichanh C A human gut microbial gene catalogue established by metagenomic sequencing Nature 2010 464 59 65 10.1038/nature08821 20203603 PMC3779803

[B29] Fang H Hedin G Rapid screening and identification of methicillin-resistant Staphylococcus aureus from clinical samples by selective-broth and real-time PCR assay J Clin Microbiol 2003 41 2894 2899 10.1128/JCM.41.7.2894-2899.2003 12843018 PMC165274

[B30] Matsuki T Watanabe K Fujimoto J Takada T Tanaka R Use of 16S rRNA gene-targeted group-specific primers for real-time PCR analysis of predominant bacteria in human feces Appl Environ Microbiol 2004 70 7220 7228 10.1128/AEM.70.12.7220-7228.2004 15574920 PMC535136

[B31] Rinttila T Kassinen A Malinen E Krogius L Palva A Development of an extensive set of 16S rDNA-targeted primers for quantification of pathogenic and indigenous bacteria in faecal samples by real-time PCR J Appl Microbiol 2004 97 1166 1177 10.1111/j.1365-2672.2004.02409.x 15546407

[B32] Million M Maraninchi M Henry M Armougom F Richet H Carrieri P Obesity-associated gut microbiota is enriched in Lactobacillus reuteri and depleted in Bifidobacterium animalis and Methanobrevibacter smithii Int J Obes (Lond) 2012 36 817 825 10.1038/ijo.2011.153 21829158 PMC3374072

[B33] Santacruz A Marcos A Warnberg J Marti A Martin-Matillas M Campoy C Interplay between weight loss and gut microbiota composition in overweight adolescents Obesity (Silver Spring) 2009 17 1906 1915 10.1038/oby.2009.112 19390523

[B34] Hartemink R Domenech VR Rombouts FM LAMVAB - A selective medium for the isolation of lactobacilli from faeces J Microbiol Methods 1997 29 77 84 10.1016/S0167-7012(97)00025-0

[B35] Ismail NA Ragab SH Elbaky AA Shoeib ARS Alhosary Y Fekry D Frequency of Firmicutes and Bacteroidetes in gut microbiota of obese and normal weight Egyptian children and adults Arch Med Sci 2010 7 501 507 10.5114/aoms.2011.23418 PMC3258740 22295035

[B36] Luoto R Kalliomaki M Laitinen K Isolauri E The impact of perinatal probiotic intervention on the development of overweight and obesity: follow-up study from birth to 10 years Int J Obes (Lond) 2010 34 1531 1537 10.1038/ijo.2010.50 20231842

[B37] Cole TJ Lobstein T Extended international (IOTF) body mass index cut-offs for thinness, overweight and obesity Pediatr Obes 2012 7 284 294 10.1111/j.2047-6310.2012.00064.x 22715120

[B38] Roelants M Hauspie R Hoppenbrouwers K References for growth and pubertal development from birth to 21 years in Flanders, Belgium Ann Hum Biol 2009 36 680 694 10.3109/03014460903049074 19919503

[B39] Koch FC **Zentralbl.** Bakteriol. Parasitenkd Abt I Orig 1942 149 122

[B40] Murray PR Rosenthal KS Kobayashi GS Pfaller MA Anaerobic gram-negative bacilli Medical Microbiology 2002 St. Louis: Elsevier Mosby 354 358

[B41] Ellner PD Stoessel CJ Drakeford E Vasi F A new culture medium for medical bacteriology Tech Bull Regist Med Technol 1966 36 58 60 5908212

[B42] Rada V Petr J A new selective medium for the isolation of glucose non-fermenting bifidobacteria from hen caeca J Microbiol Methods 2000 43 127 132 10.1016/S0167-7012(00)00205-0 11121611

[B43] Mariat D Firmesse O Levenez F Guimaraes V Sokol H Dore J The Firmicutes/Bacteroidetes ratio of the human microbiota changes with age BMC Microbiol 2009 9 123 10.1186/1471-2180-9-123 19508720 PMC2702274

[B44] Breiman L Random forests Mach Learn 2001 45 5 32 10.1023/A:1010933404324

